# The role of the ERK signaling pathway in promoting angiogenesis for treating ischemic diseases

**DOI:** 10.3389/fcell.2023.1164166

**Published:** 2023-06-22

**Authors:** Yue-Yue Song, Dan Liang, De-Kun Liu, Lin Lin, Lei Zhang, Wen-Qing Yang

**Affiliations:** ^1^ Innovation Institute of Traditional Chinese Medicine, Shandong University of Traditional Chinese Medicine, Jinan, China; ^2^ College of Traditional Chinese Medicine, Shandong University of Traditional Chinese Medicine, Jinan, China; ^3^ Shandong Province Cardiovascular Disease Chinese Medicine Precision Diagnosis Engineering Laboratory, Shandong University of Traditional Chinese Medicine, Jinan, China

**Keywords:** angiogenesis, ischemic diseases, extracellular signal-regulated kinase, vascular endothelial growth factor, drug therapy

## Abstract

The main treatment strategy for ischemic diseases caused by conditions such as poor blood vessel formation or abnormal blood vessels involves repairing vascular damage and encouraging angiogenesis. One of the mitogen-activated protein kinase (MAPK) signaling pathways, the extracellular signal-regulated kinase (ERK) pathway, is followed by a tertiary enzymatic cascade of MAPKs that promotes angiogenesis, cell growth, and proliferation through a phosphorylation response. The mechanism by which ERK alleviates the ischemic state is not fully understood. Significant evidence suggests that the ERK signaling pathway plays a critical role in the occurrence and development of ischemic diseases. This review briefly describes the mechanisms underlying ERK-mediated angiogenesis in the treatment of ischemic diseases. Studies have shown that many drugs treat ischemic diseases by regulating the ERK signaling pathway to promote angiogenesis. The prospect of regulating the ERK signaling pathway in ischemic disorders is promising, and the development of drugs that specifically act on the ERK pathway may be a key target for promoting angiogenesis in the treatment of ischemic diseases.

## 1 Introduction

Ischemic disease is one of the diseases that pose a serious threat to human health. Among these, ischemic heart disease (IHD) is considered the leading cause of deaths worldwide. Data show that ischemic heart disease caused 9.44 million deaths in 2021 (The Global Burden of Cardiovascular Disease, 2023). In 2019, stroke remained the second leading cause of deaths worldwide, with ischemic stroke accounting for 61.4% of all stroke incidents (GBD, 2019 Stroke Collaborators, 2021). It is a series of diseases caused by ischemia and hypoxia, such as insufficient blood perfusion and blocked reflux, which leads to tissue and organ dysfunction, with vascular damage as the main lesion. Blood vessels create a vast network within the body, supplying oxygen and nutrients, nourishing all body tissues and organs, and providing access to immune monitoring (Eelen et al., 2020). Angiogenesis is the natural response to a reduction in blood supply to vital organs of the body. Cytokines and other molecules transform the local microenvironment, provide an angiogenic environment, promote the generation of capillary networks in ischemic tissues, establish collateral circulation in ischemic organs, restore blood perfusion in ischemic regions, and improve the survival status of the organism (Zhang and Chen, 2021). Abnormalities in the vascular system function, which cause organs and tissues to become ischemic, can lead to development of various diseases, including ischemia in the cardiovascular system and cerebrovascular system and lower limb arterial ischemia. Therefore, angiogenesis is critical for the pathological repair of ischemic diseases.

The concept of angiogenesis was first introduced as the Folkman hypothesis in the 1970s (Folkman, 1971). Angiogenesis is the process of formation of new vascular branches and capillary plexuses from the original vascular structure in the form of sprouting or in-fill that is regulated by several factors (Brem and Tomic-Canic, 2007). Early increase in vascular permeability; degradation of the extracellular matrix; proliferation, migration, and differentiation of endothelial cells; and formation of vascular lumen-like structures are all part of angiogenesis (Li et al., 2007). Many factors are involved in angiogenesis, such as the vascular endothelial system, angiogenic growth factors, shear stress of blood flow, extravascular matrix, and protein hydrolase system, among which endothelial cell proliferation, migration, and remodeling are the fundamental pathways (Emanueli and Madeddu, 2001). Under normal physiological conditions, the vascular system delivers oxygen and nutrients, removes waste, and transports immune cells to tissues and organs (Johnson et al., 2019). Insufficient angiogenesis leads to circulatory disorders and tissue necrosis in the body, producing a series of pathological changes. When the body is in an ischemic state, there is a reduction in glucose and oxygen supply and excessive accumulation of metabolic waste. At this time, the body starts self-regulation, secretes growth factors that promote angiogenesis, and creates a suitable microenvironment for angiogenesis (Carmeliet, 2003). It can effectively alleviate the progression of ischemic disease by promoting angiogenesis around the ischemic tissue and establishing collateral circulation to improve blood supply. However, excessive angiogenesis can result in the abnormal growth of pathological tissues and destroy normal physiological tissues (Li S. et al., 2019).

The extracellular signal-regulated kinase (ERK) pathway is required for angiogenic sprouting and lumen formation and has several members, among which ERK1 and ERK2 can be activated by phosphorylation and regulate the expression of various apoptotic factors, promote cell growth and proliferation (Guo et al., 2020), stabilize the vascular structure, and participate in the angiogenic process. ERK3 is thought to be a negative regulator of cell proliferation (Julien et al., 2003). However, the exact biological role of ERK4 remains unknown. ERK5 controls embryonic angiogenesis by negatively regulating vascular endothelial growth factor (VEGF) in response to extracellular stimuli such as hypoxia and fluid shear (Jiang and Han, 2017). When activated, the ERK signaling pathway promotes abnormal cell proliferation and differentiation, enhances angiogenic capacity, and regulates angiogenesis by regulating the cell cycle, thereby improving local microcirculation, restoring blood supply to ischemic regions, and slowing the progression of ischemic disease (Shin et al., 2016). Based on the role of the ERK signaling pathway in angiogenesis, there is evidence that inhibiting the ERK signaling pathway can prevent angiogenesis (Wang et al., 2022). Furthermore, ERK signaling is critical for maintaining vascular integrity in resting endothelial cells (ECs) (Ricard et al., 2019), which demonstrates the central role of the ERK signaling pathway in regulating cellular functions involved in promoting angiogenesis.

Previously, the therapeutic significance of angiogenesis was mainly focused on inhibiting the growth of new blood vessels and delaying the spread of cancer (Lian, 2004). The induction of new blood vessel growth in ischemic diseases caused by abnormal vascular system function is currently receiving considerable attention. In this review, we summarize the mechanisms by which the ERK pathway regulates angiogenesis in ischemic diseases to provide a foundation for future research.

## 2 ERK signaling pathway and angiogenesis

The ERK signaling pathway, also known as the Ras–Raf–MEK–ERK signaling pathway, is one of the classical MAPK signaling pathways and follows the tertiary enzymatic cascade reaction of MAPKs, which is upstream activating protein → kinase of MAPK kinase (MAPKKK) → MAPK kinase (MAP-KK) → MAPK, and is involved in regulating biological functions such as cell proliferation, differentiation, and apoptosis (An et al., 2016; Liang et al., 2020). ERK is a signal transduction protein that transmits mitogenic signals and is typically located in the cytoplasm. The ERK family has five subfamilies, ERK1 to ERK5, of which the ERK1/2 signaling pathway is the most classical one. ERK1/2 catalyzes the phosphorylation of hundreds of cytoplasmic and nuclear substrates, including regulatory molecules and transcription factors. The expression of various apoptotic factors is controlled by phosphorylation responses to promote cell growth and proliferation (Roskoski, 2012; Guo et al., 2020). Stimulatory factors such as cytokines activate the ERK1/2 signaling pathway by binding to the receptor to phosphorylate ERK1/2. Activated ERK1/2 enters the nucleus and regulates transcription factor activity and gene expression by controlling various substrates such as cytoskeletal protein (CAP), phosphorylated transcription factor, and ribosomal S6 protein kinase (RSK), which then regulate the cell cycle to promote cell proliferation, differentiation, and apoptosis (Boulton et al., 1991; Anjum and Blenis, 2008). ERK3 and ERK4 are structurally related atypical MAPKs that have key differences only in the C-terminal extension, and both act synergistically to activate the protein kinase MAPK-activated protein kinase 5 (MK5) (Kant et al., 2006). The biological function of ERK4 is currently unknown; however, ERK3 is thought to negatively regulate cell proliferation (Julien et al., 2003). A vast range of mammalian tissues abundantly express ERK5; the C-terminus, which is 41 amino acids long and can be autophosphorylated, is part of the ERK5 structural domain. Truncation of the C-terminus can increase ERK5 activity, which causes ERK5 activation and nuclear translocation through cascading double phosphorylation, and thereby activates downstream factors, regulates gene transcription and expression, eventually causes a series of cellular responses (Regan et al., 2002; Nithianandarajah-Jones et al., 2014), and participates in the formation of the vascular system.

**FIGURE 1:**
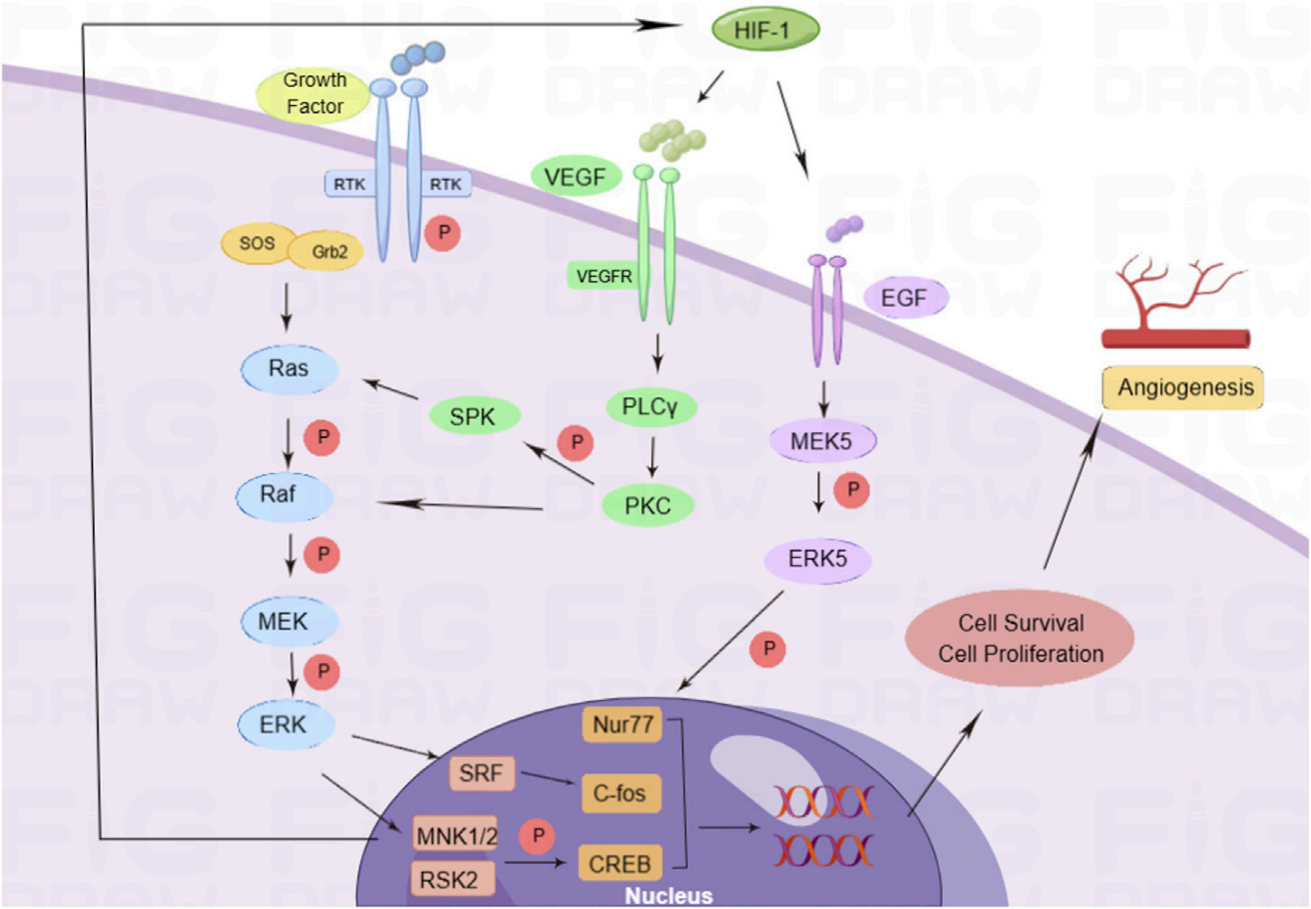
Pro-angiogenic mechanism of the ERK signaling pathway. Growth factors activate the cascade of Ras/Raf/ERK signaling pathways by activating RTK, and phosphorylated ERK enters the nucleus for transcription. In addition, during hypoxia, VEGF binds to its receptor VEGFR and activates PKC to activate the Ras/Raf/ERK signaling pathways. At the same time, hypoxia can promote EGF expression, activate ERK5 to interact with nuclear factors, regulate cell proliferation and migration, and promote angiogenesis.

Angiogenesis is controlled by complex intercellular, cell-matrix, and intracellular signaling events, and also by multiple angiogenic factors. They stimulate EC proliferation, migration, and tube formation by activating specific receptors that regulate downstream cytoplasmic and nuclear signaling events (Suzuma et al., 2000; Babaei et al., 2003; Naik et al., 2003). Growth factors can activate intracellular tyrosine kinase (RTK) by binding to the receptor on the cell membrane and then combining with the Src homology 2 (SH2) domain of the cytokine receptor-binding protein 2 (Grb2). Simultaneously, the SH2 domain combines with the ornithine conversion factor, son of sevenless (Sos), to activate the rat sarcoma virus (RAS) protein (Schaper and Ito, 1996; Zou et al., 2020). Activated Ras can activate Raf and phosphorylate downstream MEK; p-MEK can activate ERK phosphorylation, promote the expression of serum response factor (SRF), MAP kinase-interacting serine/threonine-protein kinase 1 and 2 (MNK1/2), and RSK2; activate c-fos and cAMP-response element-binding protein (CREB) transcription factors; regulate cell transcription and translation; promote cell proliferation (Asha et al., 2003); trigger the cascade amplification of the Ras/Raf-1/ERK pathway; and affect the occurrence and development of angiogenesis. Among them, VEGF is a highly specific angiogenic factor that promotes the formation of blood vessels in the body. VEGF activates phospholipase C (PLC) by binding to VEGFR on the cell membrane γ and promotes the expression of protein kinase C (PKC). On one hand, it phosphorylates downstream sphingosine kinase (SPK) and activates the RAS protein, while on the other hand, it directly activates RAF-1 and the ERK pathway (Tarnawski and Ahluwalia, 2021). VEGF expression in ECs is increased under the stimulation of hypoxia. The ERK signaling pathway can enhance the effect of hypoxia-inducible factor-1 (HIF-1) and promote the expression of VEGF, thus enhancing the transcription and translation of VEGF and improving the stability of VEGF mRNA (Zhao, 2005). HIF-1 promotes the expression of epidermal growth factor (EGF), activates MEK5, phosphorylates ERK, increases the activity of nuclear receptor subfamily 4 group A member 1 (Nur77) transcription factor, and promotes cell proliferation (Yoo et al., 2007) (Figure 1). The specific binding of VEGF and membrane receptors can activate the ERK signaling pathway and regulate cell proliferation. The activated ERK signaling pathway can promote the expression of VEGF under the stimulation of HIF-1. The interaction between VEGF and the ERK signaling pathway is involved in the regulation of angiogenesis.

## 3 The role of the ERK signaling pathway in ischemic diseases through mediating angiogenesis

Ischemic diseases, in which circulatory disorders and tissue necrosis are the main pathological changes, include IHD, ischemic cerebrovascular disease, and peripheral arterial diseases. Angiogenesis is a hypoxia-adaptive tissue response to ischemic diseases. Ischemic areas and hypoxia promote the formation of nutrient collateral circulation vessels around the blocked or narrowed vessels, increasing the number of capillaries and blood flow, thus improving ischemia and hypoxia (Koyasu et al., 2018; Esser et al., 2019). The ERK signaling pathway mediates angiogenesis by controlling cell activity and the expression of angiogenic factors (Simons et al., 2016). The ERK signaling pathway improves local microcirculation and restores blood supply to ischemic regions by regulating angiogenesis to alleviate ischemic disease progression.

### 3.1 ERK signaling pathway promotes angiogenesis to improve myocardial ischemia

**FIGURE 2:**
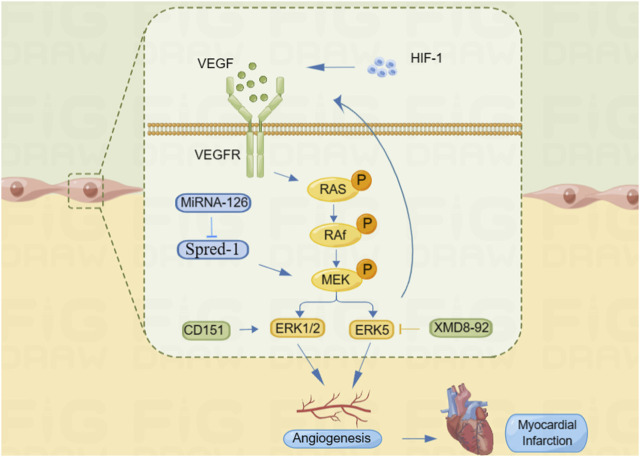
ERK signaling pathway promotes angiogenesis to improve myocardial ischemia. HIF-1 can promote VEGF expression, and VEGF binds to its receptor VEGFR and activates the Ras/Raf/ERK signaling pathway. MiRNA-126 promotes MEK phosphorylation by inhibiting Spred-1 and activates the ERK signal pathway. CD151 can directly activate the ERK1/2 signaling pathway and regulate angiogenesis. In addition, XMD8-92 can inhibit the ERK5 signaling pathway and affect the secretion of VEGF, thus regulating angiogenesis.

Restoration of blood supply after myocardial ischemic injury is an important event for tissue regeneration and repair, and angiogenesis is essential for restoring blood perfusion to the ischemic myocardium. Appropriate perfusion and vascular integrity are crucial for maintenance of myocardial homeostasis (Hilfiker-Kleiner et al., 2005). One of the popular topics in the field of fundamental cardiovascular research in recent years is the application of vascular regeneration for IHD (Xiao and Chi, 2009). Through the elevation of VEGF and its receptor expression in IHD, ERK enhances EC proliferation, migration, and the creation of tube-like structures, which also promote angiogenesis and improve myocardial ischemia (Shi et al., 2020). The VEGF/VEGFR (VEGF receptor) system is further activated by hypoxic stimuli through the activation of HIF-1. VEGF and its receptors either directly or indirectly activate signaling pathways such as Ras/Raf-MEK/ERK and endothelial nitric oxide synthase (eNOS)/nitric oxide (NO) to promote vascular neovascularization and alleviate ischemia (Liu et al., 2009). According to Liu et al. (2015), CD151 increases EC migration by activating the ERK1/2 signaling pathway, which is involved in the upregulation of VEGF expression, thereby promoting microangiogenesis, increasing collateral circulation in the ischemic and hypoxic myocardium, and improving the survival of cardiomyocytes. Upregulation of miR-126, a negative regulator of the ERK pathway, has been reported to be involved in the regulation of the pro-angiogenic kinase phosphorylated ERK (p-ERK) (Meng et al., 2012), inhibition of sprout-related EVH1 domain-containing 1 (Spred-1) expression, and activation of the ERK/VEGF pathway. Increased expression of VEGF and its receptors VEGFR-1 and VEGFR-2 in cardiomyocytes participates in myocardial revascularization in the ischemic myocardium, which improves myocardial reperfusion (Hedman et al., 2003). In addition, the phosphorylation level of ERK5 significantly increases after activation under hypoxia, and inhibition of ERK5 phosphorylation by XMD8-92 significantly improved EC proliferation, migration, tubular structure formation, and inhibition of VEGF secretion, thereby regulating myocardial angiogenic mechanisms (Cheng, 2013) (Figure 2). As a result, a new research area has emerged focusing on using the ERK signaling pathway to promote angiogenesis in ischemic myocardial tissue to preserve heart function and reduce ischemic injury.

### 3.2 The role of the ERK signaling pathway in myocardial infarction through promoting angiogenesis

**FIGURE 3:**
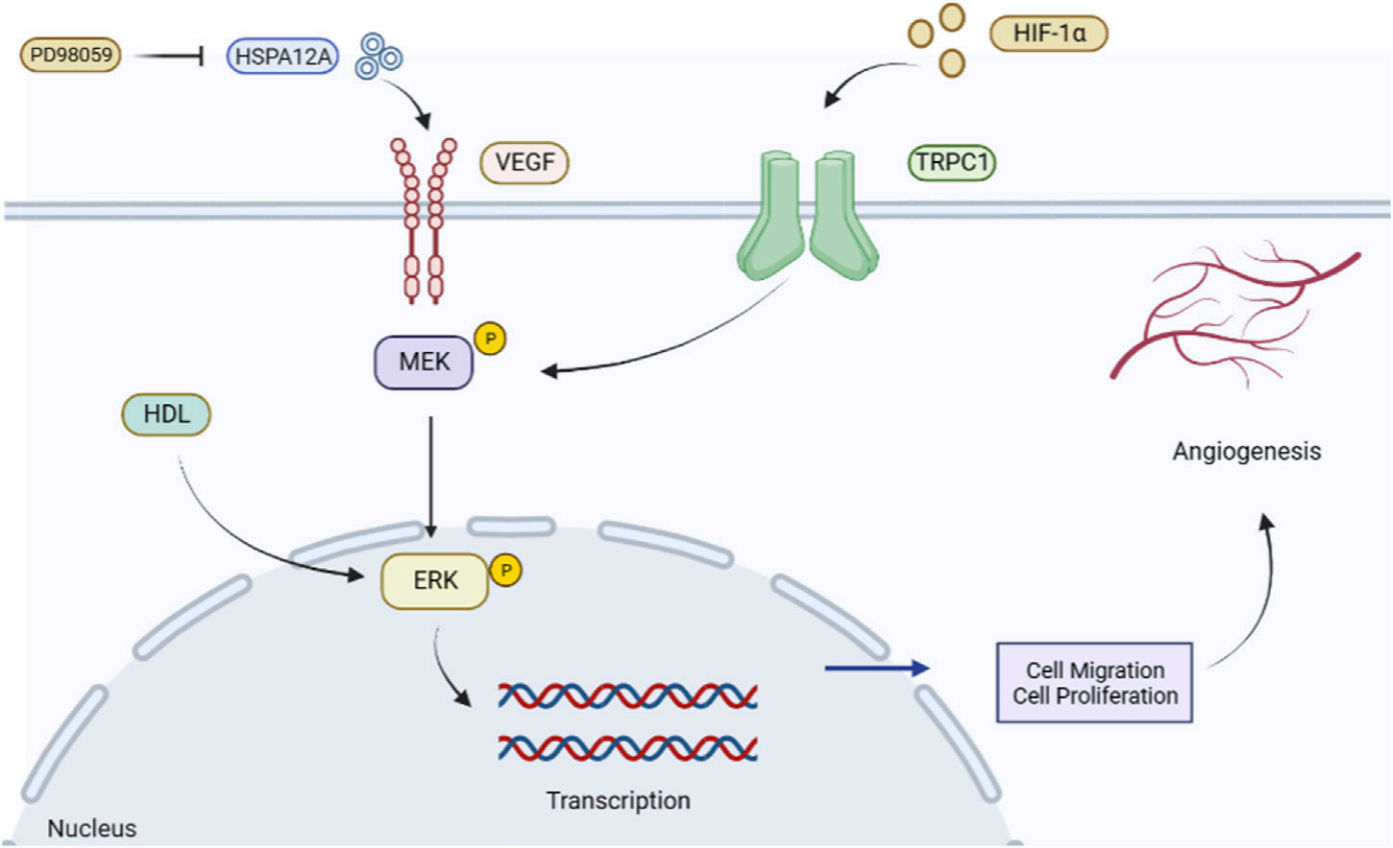
Role of the ERK signaling pathway to promote angiogenesis in myocardial infarction. HSPA12A promotes VEGF expression and activates the ERK signaling pathway. In contrast, its inhibitor PD98059 attenuates ERK phosphorylation and hinders angiogenesis. HIF-1α can improve myocardial ischemia by promoting the expression of phosphorylated MEK in TRPC1, activating the ERK signaling pathway, promoting cell proliferation and migration, regulating angiogenesis, and improving myocardial ischemia status.

Myocardial infarction is an important factor in mortality associated with IHD. Improving tissue repair and microcirculation after the acute phase of myocardial infarction remains a top priority, and promoting angiogenesis has become a widely used strategy for saving the ischemic myocardium and limiting the infarct size. After myocardial infarction, VEGF levels are enhanced in the peripheral blood as a tissue response to ischemic and hypoxic stimuli (Kranz et al., 2000). Heat shock protein 12A (HSPA12A) is a novel activator of angiogenesis that increases the expression of VEGF and the phosphorylation level of ERK in ECs, thereby mediating angiogenesis (Dai et al., 2021). The ERK inhibitor PD98059 attenuates the phosphorylation of VEGF and VEGFR2 expression through HSPA12A and attenuates angiogenic features *in vitro*; knockdown of HSPA12A in mice impairs cardiac angiogenesis after myocardial infarction and exacerbates cardiac dysfunction (Li et al., 2022). Studies have shown that hypoxic factors activate the ERK signaling pathway and HIF-1α participates in the regulation mechanism of the ERK signaling pathway (Zhang Q. et al., 2014). By constructing a mouse model of myocardial infarction, Wen et al. (2022) found that transient receptor potential canonical 1 (TRPC1) is a crucial factor in EC function and angiogenesis and that TRPC1 expression induced by HIF-1α was upregulated in myocardial infarction (MI) mice, thereby regulating the ERK signaling pathway. Knockdown of TRPC1 and HIF-1α significantly inhibited the phosphorylation of MEK and downstream ERK, thus affecting their angiogenic processes (Guo et al., 2019). Experimental studies have shown that normal high-density lipoprotein (HDL) can promote angiogenesis in human umbilical vein endothelial cells by stimulating the phosphorylation of ERK1/2, and HDL inhibits ERK1/2 phosphorylation to block angiogenesis in patients with acute myocardial infarction (Zhang et al., 2018) (Figure 3). Therefore, the ERK signaling pathway is a feasible strategy to promote new vessel formation and improve cardiac insufficiency after myocardial infarction.

### 3.3 Activation of the ERK signaling pathway promotes revascularization to improve ischemic stroke

**FIGURE 4:**
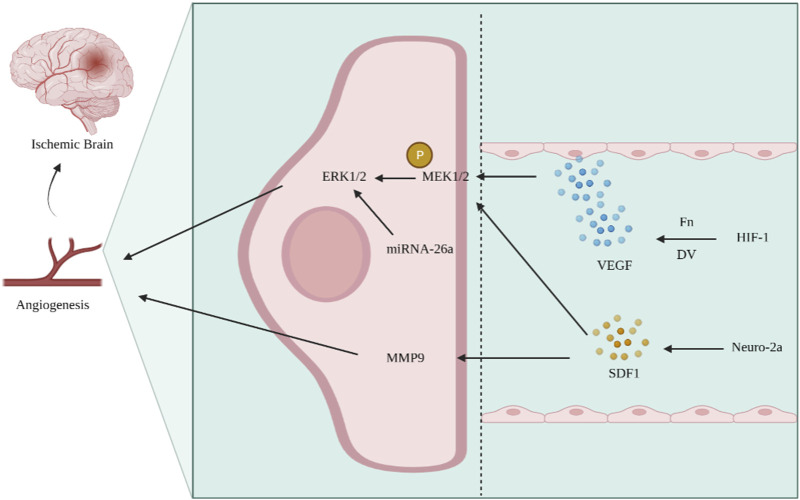
Activation of the ERK signaling pathway promotes angiogenesis and improves ischemic stroke. After cerebral ischemia and hypoxia, the components of Fn and DV increase, activating the VEGF/VEGFR system, phosphorylating MEK1/2, and activating the ERK signaling pathway. In ischemic stroke, the expression of SDF1 increases, while SDF1 can promote MEK1/2 phosphorylation and MMP9 expression, regulate angiogenesis, and improve cerebral ischemia.

Ischemic stroke is an intractable disease with high rates of morbidity, disability, and mortality. The basic principle of treatment is post-infarction side branch reconstruction, which mainly depends on angiogenesis (Zhang L. X. et al., 2021; Guan et al., 2021; Ni et al., 2021). Angiogenesis is the structural basis for tissue resistance to injury and neuronal repair in ischemic areas, and the ERK1/2 signaling pathway is involved in several pathological processes, such as angiogenesis after cerebral ischemia (Liang et al., 2018). The VEGF/VEGFR system is activated after cerebral ischemia and hypoxia, and the expression of extracellular matrix components, such as basement membrane glycan C-terminal functional region V (DV) and fibronectin (Fn), increases, which bind to their corresponding receptor integrins and release various factors, such as VEGF (Huang and Li, 2014); this promotes angiogenesis and neuroprotection through the Ras–Raf–MEK–ERK signaling pathway and activates the ERK1/2 signaling pathway. Expression of miRNA-26a increases after cerebral ischemic injury, and the ERK and AKT signaling pathways regulate the expression of VEGF and HIF-1α, thereby regulating angiogenesis in brain microvascular endothelial cells (BMECs) after cerebral infarction (Liang et al., 2018). Studies have shown that brain-derived neurotrophic factor (BDNF) can achieve pro-angiogenesis by activating the ERK1/2 signaling pathway (Chaudhry et al., 2010). Yang et al. (2013) constructed a middle cerebral artery occlusion model in rats and found that the ERK1/2 signaling pathway showed an evolutionary pattern similar to that of angiopoietin-2 (Ang-2) in promoting the angiogenic process. In cerebral arterial thrombosis, the chemotactic factor stromal-derived factor (SDF1) is released at the site of injury. After placing neuro-2a cells in SDF1 for a while, phosphorylation of ERK1/2 and expression of matrix metalloproteinase-9 (MMP-9) were significantly increased, thus promoting angiogenesis and improving brain repair after stroke (Wesley et al., 2017) (Figure 4). Therefore, the activation of the ERK signaling pathway to promote revascularization provides a new direction for improving cerebral ischemia–reperfusion and reducing neurological impairment.

### 3.4 The role of the ERK signaling pathway in limb artery ischemia through angiogenesis

**FIGURE 5:**
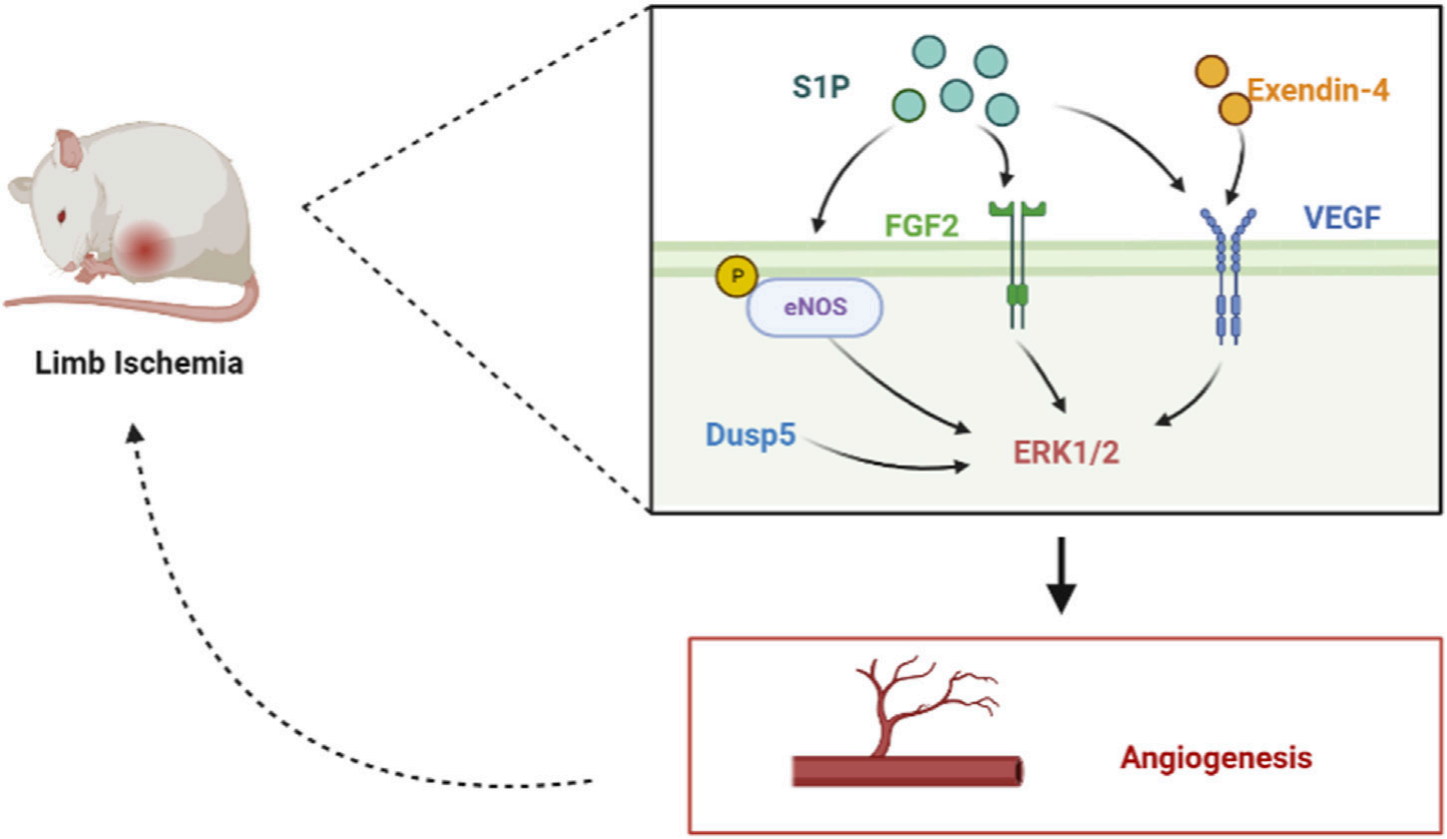
Role of the ERK signaling pathway in limb artery ischemia by mediating angiogenesis. In the hindlimb ischemia mouse model, S1P can phosphorylate eNOS, upregulate the expression of FGF2 and VEGF, and activate the ERK signal pathway. In addition, exendin-4 can upregulate VEGF and activate the ERK signal pathway. Dusp5 can directly activate the ERK1/2 signal pathway, promote angiogenesis, and improve limb ischemia.

Angiogenesis is an important strategy to improve ischemic diseases of the limbs caused by lower limb vascular occlusion. Long-term limb ischemia can lead to severe functional disabilities, and stimulating angiogenesis to treat limb ischemia has become an attractive modality. Arteriogenesis and angiogenesis are adaptive mechanisms used in limb ischemia to restore tissue perfusion (Scholz et al., 2002). The lysophospholipid mediator sphingosine-1-phosphate (S1P) plays an important role in vascular development by activating the ERK1/2 and AKT signaling pathways and increasing the phosphorylation of eNOS, which stimulates post-ischemic angiogenesis and blood flow recovery. After preparing S1P microspheres to act on a mouse hind limb ischemia model, Qi et al. (2010) found that S1P increased the phosphorylation of eNOS and the expression of multiple angiogenic factors in ischemic muscle by promoting vascular coverage of smooth muscle cells and pericytes, which in turn increased microvascular density in ischemic muscle, and eNOS phosphorylation activates the ERK1/2 signaling pathway (Urano et al., 2008), which is involved in angiogenesis and post-ischemic blood flow restoration for access to ischemic muscles to alleviate limb necrosis and dysfunction. By constructing a mouse hindlimb ischemia model, Kang et al. (2015) found that exendin-4 upregulated VEGF and elevated phosphorylated ERK-induced angiogenesis, which can effectively prevent ischemic injury in mice. Through the induction of a mouse model of hindlimb ischemia, Alleboina et al. (2019) found that dual-specific phosphatase 5 (DUSP5) is upregulated in ischemia-exposed ECs. DUSP5 regulates post-ischemic EC proliferation and angiogenesis by phosphorylating the mitogen-activated protein kinase ERK1/2 and reducing p21 protein expression. By establishing a model of surgically induced acute hindlimb ischemia in diabetic rats, Pan et al. (2013) found that the upregulation of fibroblast growth factor (FGF)-2 and FGFR1 expression activates the ERK1/2 pathway and promotes angiogenesis and ischemic reperfusion (Figure 5). Therefore, the ERK signaling pathway, which promotes angiogenesis, is a promising therapeutic strategy for the treatment of limb ischemia.

## 4 Drug interventions in angiogenesis for ischemic diseases

Currently, the modulation of angiogenesis through pharmacological interventions to regulate pathways and specific gene expression is an important therapeutic measure to delay and improve ischemic diseases. Pharmacological modulation of the ERK signaling pathway for pro-angiogenesis is a hot research topic in the treatment of ischemic diseases. Many different drug categories are used in pharmacological research on the ERK pathway (Table 1), such as traditional Chinese medicine (TCM) and natural drug components, which can delay the progression of ischemic diseases by interfering with angiogenesis.

**TABLE 1:** Summary of drug intervention angiogenesis in the treatment of ischemic diseases.

Drugs	Composition/structure	Diseases	Signal pathway	References
Tongnao Decoction	Gastrodiae Rhizoma, Uncariae Ramulus Cum Uncis, Chuanxiong Rhizoma, Arisaematis Rhizoma Preparatum, Rhodiolae Crenulatae Radix et Rhizoma, Rhizoma Anemone Altaicae, Hirudo, and Bombyx batryticatus	Ischemic stroke	VEGF/ERK signal pathway	Wang et al. (2020); Wang (2021)
Buyang Huanwu Decoction	Astragali Radix, Angelica Sinensis Radix, Paeoniae Radix Rubra, Pheretima, Chuanxiong Rhizoma, Persicae Semen, and Carthami flos	Myocardial infarction	Cav-1/VEGF/ERK signal pathway	Zhu et al. (2019); Peng et al. (2020)
Tongxinluo	Ginseng Radix et Rhizoma, Paeoniae Radix Rubra, Santali album Lignum, Dalbergiae Odoriferae Lignum, Olibanum, Ziziphi Spinosae Semen, Borneolum Syntheticum, Hirudo, Scorpio, Cicadae Periostracum, Eupolyphaga steleophaga, and Scolopendra	Myocardial infarction	MAPK/ERK signal pathway	Bai (2014); Bai et al. (2017)
Danhong injection	Salviae Miltiorrhizae Radix et Rhizoma and Carthami flos	Myocardial infarction	ERK signal pathway	Li et al. (2019b); He et al. (2019)
PFE	, 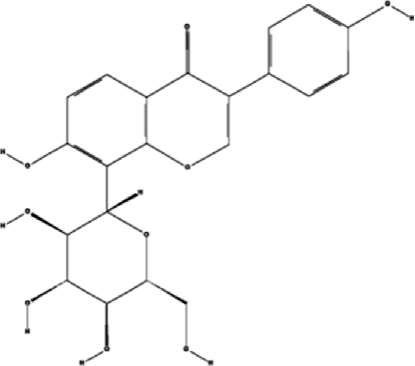	Ischemic cardiovascular disease	MEK/ERK, PI3K/AKT/eNOS, SRC/FAK, and VEGF/VEGFR2 signal pathways	Chung et al. (2010a); Zhai et al. (2021)
AS-IV	, 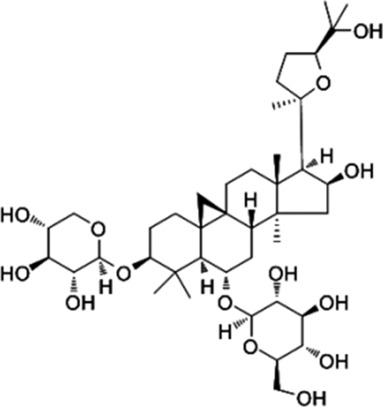	Myocardial infarction	ERK1/2 signal pathway	Wang et al. (2015)
TALH	, 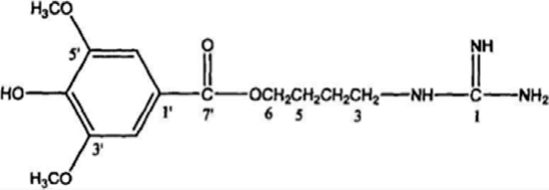	Wound ischemia	SRC/MEK/ER signal pathway	Shi et al. (2022)
Salvianolic acid B	, 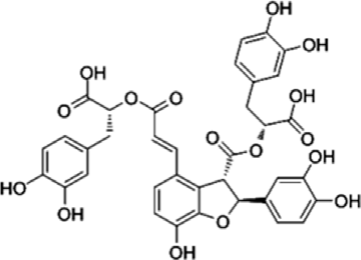	Ischemic stroke	AKT/ERK signal pathway	Li (2020)
MDG-1	, 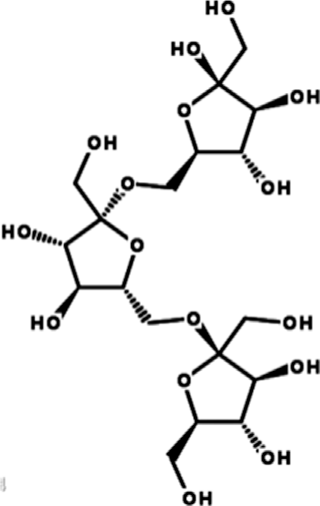	Myocardial ischemia	S1P/bFGF/Akt/ERK/eNOS signal pathway	Wang et al. (2010)
Sesamin	, 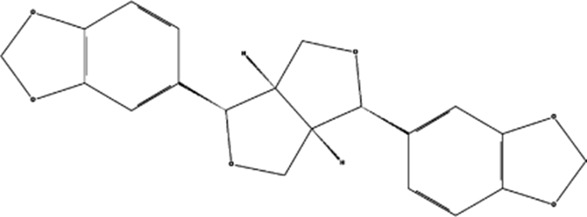	Ischemic cardio-cerebrovascular disease	MEK/ERK and P38 MAPK signal pathways	Fukuda et al. (1986); Lee et al. (2004); Nakano et al. (2006); Chung et al. (2010b)
KYZ	, 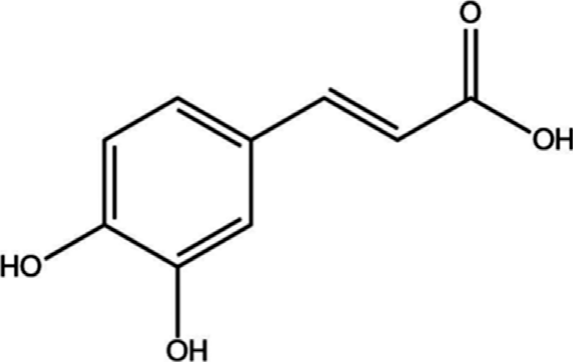	Ischemic cardio-cerebrovascular disease	PI3K/AKT and MEK/ERK signal pathways	Kong et al. (2022)
Irisin	, 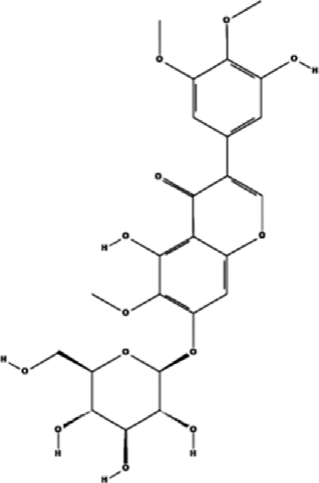	Myocardial ischemia	ERK–MAPK signal pathway	Mavria et al. (2006); Zhang et al. (2014b); Liao et al. (2019)
H_2_S	, 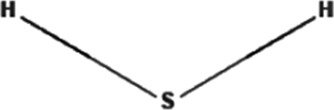	Ischemic cardio-cerebrovascular disease	AKT and ERK signal pathways	Zicola et al. (2021)
2HP-β-CD	, 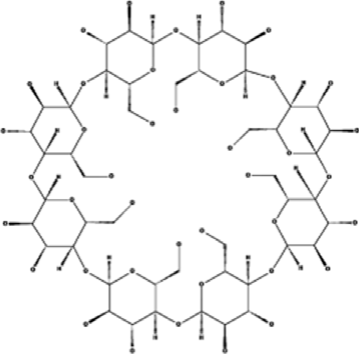	Limb ischemia	VEGF-A, PDGF-BB, TGF-β1, and ERK signal pathway	Qi et al. (2015)
PD98059	, 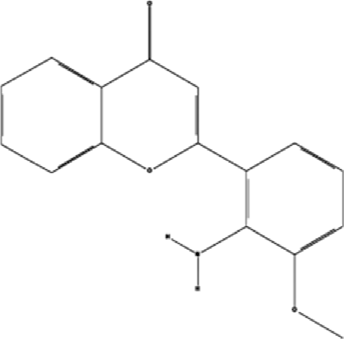	Colorectal cancer	ERK signal pathway	Hao et al. (2014)
Sorafenib	, 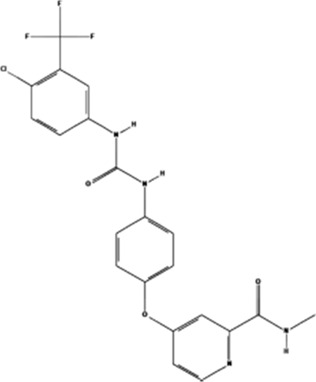	Cancer of the liver	ERK signal pathway	Wang (2017)

### 4.1 TCM prescriptions

#### 4.1.1 Tongnao Decoction

Tongnao Decoction is prepared with eight Chinese herbs, i.e., Tianma, Uncaria rhynchophylla, Chuanxiong, Nanxing, Rhodiola, Altai Anemone Root, Hirudo, and Jiangcan. According to recent research, Tongnao Decoction enhances cerebral microcirculation, repairs the vascular endothelium, and protects brain cells (Wang, 2021). Studies have shown that Tongnao Decoction can promote vascular EC proliferation, migration, and lumen formation *in vitro* and significantly upregulate VEGF expression in ECs *in vivo*. The VEGF-initiated signaling pathway phosphorylates ERK1/2 to activate the signaling cascade through the Raf/MEK pathway to promote angiogenesis (Wang et al., 2020). Tongnao Decoction is an effective TCM formula for improving neurological function. Activating the ERK signaling pathway has a pro-angiogenic effect that may be the key to accelerating neurological recovery in ischemic stroke.

#### 4.1.2 Buyang Huanwu decoction

The Buyang Huanwu Decoction is obtained from the Correction of Errors in Medical Works and consists of seven herbs: Huangqi, Shengguiwei, Chishao, Pberetima, Chuanxiong, Taoren, and Honghua. Modern studies have shown that the Buyang Huanwu Decoction has antioxidant, anti-inflammatory, anti-thrombotic, anti-apoptotic, and pro-vascular renewal properties; corrects ion metabolism disorders; and improves the metabolic capacity of brain tissue (Peng et al., 2020). Studies have indicated that the Buyang Huanwu Decoction stimulates VEGF and VEGFR2 expression by increasing the expression of caveolin-1, increasing ERK phosphorylation, regulating EC angiogenic activity, and stimulating angiogenesis through the Cav-1/VEGF/ERK signaling pathway and protects the heart muscle in mice with myocardial infarction (Zhu et al., 2019).

#### 4.1.3 Tongxinluo

Tongxinluo is composed of 12 Chinese herbs: Ginseng, Chishao, sandalwood, Jiangxiang, Olibanum, Suanzaoren, Bingpian, Hirudo, scorpion, Chantui, woodlouse, and Scolopendra, which regulate qi, promote circulation, relieve pain, activate blood circulation, and resolve blood stasis. Modern research has shown that it can reduce ischemia–reperfusion injury and regulate vascular endothelial function (Bai, 2014). Studies indicate that Tongxinluo can increase VEGF expression and HIF-1α expression activity by enhancing the phosphorylation of ERK and AKT, thereby increasing angiogenesis and improving cardiac function after myocardial infarction (Bai et al., 2017).

#### 4.1.4 Danhong injection

Danhong injection, extracted from Salvia miltiorrhiza and Honghua, is effective in activating blood circulation, resolving blood stasis, and promoting vascular renewal, which can play a role in the treatment of ischemic stroke by improving blood supply and microcirculation in the brain and promoting vascular neovascularization (He et al., 2019). By creating a mouse model of myocardial infarction, Li S. N. et al. (2019) discovered that Danhong injection may upregulate the expression of miR-126, activate the ERK pathway, promote the expression of VEGF-A, and contribute to the regulation of post-infarction angiogenic effects.

### 4.2 Natural pharmaceutical ingredients

#### 4.2.1 Puerariae Flos

Puerariae flos (PFE) is an extract of the Chinese herbal medicine Gegen that promotes neovascularization, EC migration, proliferation, and tube formation (Chung et al., 2010a). Studies have shown that PFE activates ERK, Akt, eNOS, NO production, p38, Src, and focal adhesion kinase (FAK) but does not increase the expression of VEGF. The inhibition of ERK, Akt, and eNOS inhibited PFE-induced angiogenic events, while the inhibition of p38 and Src activity blocked PFE-induced EC migration. By activating the MEK/ERK, phosphatidylinositol 3-kinase/Akt/eNOS, and Src/FAK-dependent pathways, PFE directly stimulates angiogenesis in the treatment of ischemic cardiovascular disease (Zhai et al., 2021).

#### 4.2.2 Astragaloside IV

Astragaloside IV (AS-IV) is an active ingredient isolated and purified from Huangqi and has the function of protecting endothelial function and promoting vascular regeneration. Studies have shown that AS-IV promotes cell proliferation and capillary lumen formation in human umbilical vein endothelial cells (EA-hy926); upregulates VEGF-related mRNA and protein expression; generates pro-angiogenic biological activity by activating the ERK1/2 signaling pathway to form p-ERK, which is also a regulating factor of VEGF (Wang et al., 2015); and establishes collateral circulation for myocardial infarcted tissue, thereby restoring blood supply.

#### 4.2.3 Total alkaloid fraction from Leonurus japonicus Houtt

The total alkaloid fraction from Leonurus japonicus Houtt (TALH) is one of the main components of the Chinese herb Yimoucao, which promotes EC proliferation, migration, and tube formation. Studies have indicated that TALH can increase VEGF expression and enhance the expression of SRC, MEK1/2, and ERK1/2 genes. The phosphorylation levels of these proteins regulate the SRC/MEK/ERK pathway to induce angiogenesis and restore blood flow to ischemic areas (Shi et al., 2022).

#### 4.2.4 Salvia miltiorrhiza polyphenolic acid B

Salvia miltiorrhiza polyphenolic acid B is one of the main chemical components of the Chinese medicine Danshen and has the effect of improving microcirculatory disorders and promoting vascular regeneration. Studies have indicated that Salvia miltiorrhiza polyphenolic acid B can increase the number of cerebrovascular ECs; promote the generation of EC tubular structures; increase the expression of angiogenesis-related markers VEGFA, VEGFR2, ANGPT1, ANGPT2, and Tie2; and further upregulate the phosphorylation levels of p-AKT and p-ERK1/2, thus promoting the angiogenic effects of ECs (Li, 2020) and restoring blood flow after ischemic stroke.

#### 4.2.5 β-d-fructan

β-d-fructan (MDG-1), one of the components of the traditional Chinese medicine Maidong, protects cardiomyocytes and microvascular endothelial cells (HMEC-1) from cell death induced by oxygen–glucose deprivation (OGD). *In vitro*, MDG-1 promotes HMEC-1 differentiation into capillary tube-like structures, acts as a chemotactic agent in cell migration assays, and promotes angiogenesis in the ischemic myocardium. Additionally, MDG-1 upregulates the expression of sphingosine kinase 1 and S1P receptor 1. Meanwhile, bFGF expression and Akt, ERK, and eNOS phosphorylation in HMEC-1 cells are induced by both MDG-1 and S1P. Induction of the cytoprotective and pro-angiogenic effects of S1P1 and bFGF through the S1P/bFGF/Akt/ERK/eNOS signaling pathway protects cardiomyocytes and HMEC-1 from ischemia-induced cellular injury (Wang et al., 2010) and improves the myocardial ischemic status.

#### 4.2.6 Sesamin

The natural product sesamin is considered a potent antioxidant (Fukuda et al., 1986). Studies have shown that in animal models, sesamin increases angiogenesis *in vitro* through EC proliferation, migration, tube formation, and neovascularization. This compound activates a variety of angiogenic signaling regulators such as ERK, Akt, eNOS, NO production, and P38 MAPK (Lee et al., 2004; Nakano et al., 2006). The MEK inhibitor PD98059 and the PI3K inhibitor wortmannin specifically inhibited sesamin-induced activation of the ERK and Akt/eNOS pathways, reduced angiogenic events, and had high specificity for MEK-/ERK-dependent cell proliferation and migration and PI3K/Akt-mediated tube formation. In addition, the compound did not induce vascular permeability, and the expression of intercellular adhesion molecule 1 (ICAM-1) and VCAM-1, which are markers of vascular inflammation, was not upregulated (Chung et al., 2010b). Sesamin is particularly important for the treatment of ischemic cardiovascular system diseases and tissue regeneration because it activates ERK and other dependent pathways that stimulate angiogenesis *in vitro* and *in vivo* without increasing vascular inflammation.

#### 4.2.7 Isopropyl caffeic acid

Isopropyl caffeic acid (KYZ) is a constituent of the Chinese herbal medicine Salvia miltiorrhiza, which was chemically synthesized by Professor Xiaohui Zheng of Northwest University. Studies have shown that KYZ induces human umbilical vein endothelial cells (HUVECs) to release VEGF factors, upregulates the expression of VEGFR2, upregulates the phosphorylation levels of MEK1/2 and ERK1/2, activates the PI3K/AKT and MEK/ERK signaling pathways, enhances cell migration ability and lumen formation, and promotes vascular neogenesis (Kong et al., 2022), which has a promising future in ischemic cardiovascular and cerebrovascular diseases.

### 4.3 Others

#### 4.3.1 Irisin

Irisin is a myokine that protects against lung and heart damage. Studies have shown that in HUVECs, irisin binds directly to the cell membrane, enhances ERK phosphorylation, and induces HUVEC migration by activating the ERK signaling pathway (Zhang Y. et al., 2014; Liao et al., 2019). ERK–MAPK signaling promotes angiogenesis by enhancing EC survival and sprouting (Mavria et al., 2006) and improves ischemic myocardial reperfusion function.

#### 4.3.2 Hydrogen sulfide

Hydrogen sulfide (H_2_S) is an effective vasodilator that plays an important role in ischemia/reperfusion injury of the cardiovascular system. Studies have shown that H_2_S increases the expression of VEGF and Ang-1 and enhances angiogenesis in the peri-infarct region by promoting the phosphorylation of AKT and ERK to restore reperfusion and improve neurological function after ischemic stroke in a rat middle cerebral artery occlusion (MCAO) model (Zicola et al., 2021).

#### 4.3.3 2-Hydroxypropyl-β-cyclodextrin

A new angiogenic molecule, 2-hydroxypropyl-β-cyclodextrin (2HP-β-CD), increases the contents of VEGF-A and platelet-derived growth factor BB (PDGF-BB) peptides in HUVECs and bFGF peptides in human aortic smooth muscle cells (HASMCs). NO has been shown to be a critical mediator of angiogenesis, and 2HP-β-CD stimulates HUVEC proliferation and migration in an eNOS-/NO-dependent manner, whereas NO is only involved in HASMC proliferation, but not migration. The unilateral hindlimb ischemia mouse model treated with 2HP-β-CD increased the expression of VEGF-A, PDGF-BB, and transforming growth factor-1 (TGF-1) in ischemic muscle; stimulated protein kinase B and ERK; improved eNOS phosphorylation in ischemic muscle; increased ischemic muscle microvascular density; promoted blood flow recovery; and stimulated smooth muscle cell coverage of blood and stabilized blood vessels (Qi et al., 2015), providing a basis for functional recovery in patients with limb ischemia.

#### 4.3.4 PD98059

PD98059 is a specific inhibitor of MEK. MEK is an important component of the ERK signal transduction pathway. PD98059 prevents its phosphorylation by binding to the non-activated form of MEK1/2, thereby inhibiting the phosphorylation of ERK1/2, blocking the growth and proliferation of cells, and the formation of EC tubules (Hao et al., 2014). This shows that PD98059 affects the biological activity of cells by inhibiting the ERK signaling pathway, thus inhibiting angiogenesis.

#### 4.3.5 Sorafenib

Sorafenib, a bis-aryl urea compound, was initially developed as an Raf-1 inhibitor with strong activity against other protein kinases. It can inhibit angiogenesis-related kinases such as the arginine kinase receptor that promotes angiogenesis, thereby affecting angiogenesis. After treatment with sorafenib, hepatoma cells upregulate miRNA-375 expression and downregulate the expression of platelet-derived growth factor C (PDGFC), inhibit the phosphorylation of its downstream signaling pathways ERK1/2, AKT, and play a role in inhibiting angiogenesis (Wang, 2017).

## 5 Summary and outlook

The relationship between angiogenesis and the treatment of ischemic diseases has received considerable attention. Pro-angiogenesis refers to the stimulation of exogenous vascular growth factors or other therapeutic methods to induce new blood vessels in peri-ischemic tissues and create new compensatory collateral circulation to enhance blood supply to the ischemic and peri-ischemic tissues. Angiogenesis is the primary adaptive pathophysiological response in ischemic disease. In recent years, relevant studies have reported that drug interventions can balance excessive and insufficient angiogenesis by regulating the expression of specific pathways and genes, indicating that promoting angiogenesis is an important means of treating ischemic diseases and prolonging the life of patients. At present, research on the effect of single and compound Chinese medicines on the ERK signaling pathway and angiogenesis, which explores the mechanism of action of TCM on angiogenesis, provides a new scientific basis for further exploring the mechanism of action of TCM in treating ischemic diseases.

Ischemic disease is characterized by reduced blood supply and insufficient supply of oxygen and nutrients. Therefore, angiogenesis and blood supply reconstruction have become the key to the treatment of ischemic diseases. The use of angiogenesis in the treatment of ischemic diseases has become one of the hotspots in the research field (Beltrán-Camacho et al., 2021). Angiogenesis has the function of tissue repair and regeneration, which is beneficial to the recovery of tissue and organ function. A significant amount of data show (Silvestre, 2012; Zhang B. L. et al., 2021) that therapeutic angiogenesis and promoting blood flow recovery therapy are of great significance for patients with ischemic diseases. However, because the process of angiogenesis is relatively slow and cannot provide enough blood for ischemic tissue, there are some limitations in the treatment of ischemic diseases, and the mechanism of improving the efficiency of angiogenesis needs to be further studied. Promoting the strategy of revascularization of ischemic tissue by stimulating angiogenesis-related signal pathways by drugs has become an important direction in the treatment of ischemic diseases.
